# Aging and GABA

**DOI:** 10.18632/aging.101480

**Published:** 2018-06-13

**Authors:** Koen Cuypers, Celine Maes, Stephan P. Swinnen

**Affiliations:** 1 Movement Control and Neuroplasticity Research Group, Department of Movement Sciences, Group Biomedical Sciences, KU Leuven, Leuven, Belgium; 2 Leuven Brain Institute (LBI), KU Leuven, Leuven, Belgium

**Keywords:** aging, inhibition, gamma-aminobutyric acid (GABA)

Healthy aging is associated with structural and functional alterations in the brain and declines in multiple facets of motor performance such as balance, fine motor skills and motor coordination. Inhibitory processes are essential for optimal brain function and undergo age-related alterations that may account for these behavioral deficits. Specifically, the inability to successfully modulate corticospinal excitability has been linked to declined motor performance in older adults [1]. In this regard, a key role is played by gamma-aminobutyric acid (GABA), i.e. the main inhibitory neurotransmitter. To demonstrate the importance of GABA in human movement control, complementary neuroimaging as well as non-invasive brain stimulation techniques can be employed to unravel age-related alterations in inhibitory function.

On the one hand, GABA levels can be regionally quantified *in vivo* using magnetic resonance spectroscopy (MRS). Multiple MRS studies point towards an age-related decline in GABA levels, correlating with degraded motor performance as well as poor cognitive functioning. In terms of measurement of age-related changes in GABA levels using MRS, a major question of interest is whether brain structure alterations need to be considered. More specifically, the identification of age-related decreases in GABA level in the brain seems to be dependent on whether loss of gray matter is considered in the quantification of GABA levels or not [2]. Besides improvements in measurement techniques, more insight into the reliability of MRS-based measures over time as well as differences in GABA levels across areas covering the cortical-subcortical territory across the lifespan is warranted. Furthermore, GABA modulation is a critical entry point for the emergence of neuroplasticity. More specifically, a reduction in GABA level is associated with training-induced motor plasticity. The question remains whether and how GABA modulation can be facilitated in the brains of older adults to promote lifelong plasticity.

Alternatively, noninvasive brain stimulation techniques (such as transcranial magnetic stimulation, TMS) provide tools to study the functional status and task-related modulation of two major receptor subtypes, i.e. GABAA (fast acting ionotropic) and GABAB (slower acting metabotropic), mediating inhibition at shorter and longer time scales, respectively [3]. Motor evoked potentials (MEPs) provide a peripheral window into the central dynamic balance between inhibition and facilitation. Although most studies demonstrate age-related declines in both GABAA and GABAB-mediated inhibition within the primary motor cortex (M1), a minority report no age-related differences or even increased inhibition in older as compared to younger adults [4]. Interestingly, the ability to modulate GABAA-ergic inhibition appears related to motor performance in older adults [5]. Notably, MRS-obtained GABA levels do not seem to correlate with TMS-obtained measures of GABAergic inhibition [4] and this requires further investigation to unravel their unique contribution to brain function and behavior.

Alternative brain stimulation techniques allow scientists to reach beyond the motor cortical network to determine inhibitory mechanisms at work across the broader cortical territory. One area that has received prominent attention concerns the use of transcranial direct or alternating current stimulation (tDCS, tACS) to probe dynamic effects on GABA level in particular brain areas and their associated cortical networks [6]. More research is warranted to establish the conditions under which stimulation effects can be obtained in older adults and how individual differences in brain status mediate these effects.

Another recent development enabling exploration of the broader cortical territory for the study of inhibitory function is TMS combined with electroencephalography (EEG). With this technique TMS-evoked EEG potentials (TEPs) can be measured, characterized by a wave with peaks at shorter and longer latencies reflecting GABAA- and GABAB-mediated inhibition, respectively. Preliminary work has shown an age-related deterioration of GABAA-mediated inhibition in the prefrontal cortex, whereas other studies have shown GABAB-mediated inhibition within M1 to increase with advancing age [5,7].

Future research is warranted to shed light on the relationship between different methodologies as well as the exact relationship between GABA-mediated processes and behavioral performance across the lifespan. In this regard, besides focusing on GABA-mediated inhibitory processes alone, a promising avenue is to also consider measures of glutamate (i.e. the main excitatory neurotransmitter) and other neurochemical compounds to elaborate on the balance between excitatory and inhibitory processes in relation to (degraded) motor performance. Furthermore, increased methodological consensus for both MRS and TMS measures of inhibition will be instrumental to allow comparisons among studies and to identify convergent aging mechanisms. We anticipate that scientists will become increasingly involved in revealing the mysteries of inhibitory function in the aging brain and its consequences for behavioral function and neuroplasticity.
